# The Leader Position of Mesenchymal Cells Expressing N-Cadherin in the Collective Migration of Epithelial Cancer

**DOI:** 10.3390/cells9030731

**Published:** 2020-03-16

**Authors:** Inés Saénz-de-Santa-María, Lucía Celada, María-Dolores Chiara

**Affiliations:** 1 Instituto de Investigación Sanitaria del Principado de Asturias, Hospital Universitario Central de Asturias, 33011 Oviedo, Spain; ines_ssantamaria@hotmail.com (I.S.-d.-S.-M.); celadalucia@hotmail.com (L.C.); 2 CIBERONC, 28029 Madrid, Spain; 3 Instituto de Oncología del Principado de Asturias, Universidad de Oviedo, 33006 Oviedo, Spain

**Keywords:** collective cell migration, N-cadherin, E-cadherin, epithelia–mesenchymal transition, cancer-associated fibroblasts

## Abstract

Understanding how heterogeneous cancer cell populations migrate collectively is of paramount importance to arrest metastasis. Here, we applied 3D culture-based approaches for in vitro modeling of the collective migration of squamous carcinoma cells and examine the impact of epithelial and mesenchymal cell interactions on this type of migration. We show that both mesenchymal N-cadherin-expressing cancer cells and cancer-associated fibroblasts cooperate in collective migration of epithelial cancer cells by leading their collective migration. This was consistent with the observed distribution of E-cadherin/N-cadherin in the human carcinoma tissues of head and neck. The presence of “leader” mesenchymal cancer cells or “leader” fibroblasts was significantly associated with metastasis development, recurrent disease and low overall disease survival in head and neck squamous cell carcinomas (HNSCC). In silico analysis of independent public datasets revealed that increased N-cadherin expression in the heterogeneous cancer tissues is associated with disease progression not only in HNSCC but also in other prevalent tumors, such as colorectal, breast and lung cancer. Collectively, our data highlight the importance of mesenchymal cells in collective cell migration and disease progression, findings that may have a broad significance in cancer, especially in those in which aberrant N-cadherin expression negatively impacts disease survival.

## 1. Introduction

Tumor metastases are responsible for as much as 90% of all cancer-related deaths, yet it remains the most poorly understood feature of cancer pathogenesis. Two distinct patterns of tumor cell invasion have been described: single-cell migration, leading to dissemination of individual tumor cells, and collective migration, resulting in multi-cellular cancer cell clusters [1,2,3].

Epithelial-to-mesenchymal transition (EMT) has been proposed as the critical mechanism for the acquisition of metastatic phenotypes by epithelial cancer cells that invade as single cells [4,5,6]. During EMT, epithelial cells disrupt tight cell–cell contacts and activate mesenchymal programs acquiring a fibroblast-like morphology with increased invasiveness and cell–stroma interactions, thus leading to the dissemination of single tumor cells. At the molecular level, a major hallmark of EMT is reduced E-cadherin expression, which results in weakened cell junctions followed by cell detachment and the onset of a single-cell mode of migration. E-cadherin loss is frequently coupled with increased N-cadherin expression, which is thought to contribute to a stroma-oriented cellular adhesion profile leading to more motile, invasive and metastatic cell phenotypes [7,8].

Besides this mode of single cell invasion, metastases can also arise from the collective invasion of tumor cells that invade as a cohesive group forming multi-cellular clusters that delaminate from the primary cell mass [3,9,10]. In contrast to single cell migration, cells that migrate collectively retain their cell–cell junctions through continuous expression of adhesion molecules, such as E-cadherin [8,11].

In recent years, it has become apparent that the plasticity of tumor and stromal cells, as well as the factors accumulating in the host because of the tumor’s presence, may also play a critical role in metastasis. Cancer cell phenotypic plasticity has been shown to play a major role in collective cell invasion such that multi-cellular cluster migration is frequently led by a subset of “leader and invasion-competent cells” that induce the collective invasion of otherwise “followers and invasion less-competent” epithelial cells [10]. More recently, it has also been reported that cancer associated fibroblasts (CAFs) may drive the collective invasion of cancer cells [12] through an intercellular physical force transmitted by heterophilic adherens junctions involving E-cadherin on the cancer cell membrane and N-cadherin on the CAF membrane [13,14]. Unfortunately, however, a clear picture in human tumor tissues suggestive of a role for CAFs as leaders of the cancer cell invasive fronts are still lacking. Moreover, the impact of that tumor phenotype on the progression of cancer disease is unknown.

Human head and neck squamous cell carcinomas (SCC) typically reveal a phenotype of collective cell movement [14]. Indeed, clustered cohort-like cancer cell dissemination appears to be highly efficient in embolizing lymphatic and blood vessels, which are accepted as a major prognostic factor for head and neck SCC [15]. Nevertheless, little is known about the complexity of collective cell invasion in this cancer type and further work is required in order to fully understand the involvement of phenotypic plasticity of tumor and stromal cells in this phenomenon. To address this issue, we set up an in vitro system for the analysis of the collective cell migration of SCC cells in 3D systems. We found that not only stromal fibroblast-like cells, whose role in collective cell migration has been previously described, but also N-cadherin-positive mesenchymal tumor cells act as leaders of the collective migration of E-cadherin-expressing epithelial tumor cells. Remarkably, these in vitro findings were found to be consistent with the topographic distribution of tumor cells expressing N-cadherin and stromal fibroblast-like cells in head and neck SCC tissues. We identified head and neck SCC tissues in which either mesenchymal cancer cells or fibroblast-like cells were located at the tips of invasive tumor nests, a phenotype that it was found associated with a low overall patient survival. These data give our findings clinical relevance and provide the basis for targeting mesenchymal-type cancer cells for personalized cancer therapy, especially in those cancers in which N-cadherin overexpression negatively impact disease survival.

## 2. Materials and Methods

### 2.1. Cell Culture

The established human squamous cell carcinoma (SCC)-derived cell lines were kindly provided by Dr R. Grenman (University Central Hospital, Turku, Finland). Cancer-associated stromal fibroblast-like cell lines were derived from surgically removed SCCs arising at the larynx (CAF1) or oral cavity (CAF3) (see Figure S1 for CAF phenotypical characterization). Normal fibroblast-like primary cultures (NF) were derived from the oral mucosa of a non-cancerous young patient. All cell lines were grown as previously described [16] and were periodically tested for human pathogens and mycoplasma infection. To avoid cross-contamination and phenotype changes, the cell lines have not been maintained in long-term cultures. All cells used in this study were maintained as frozen stocks and cultured for 2 to 4 weeks only before use in the experiments. Array CGH had been used to characterize genome-wide DNA copy number alterations in these cell lines and authenticate them. This analysis had revealed the presence of an overall pattern that is broadly consistent with the literature in head and neck squamous cell carcinomas. Authentication of these cell lines based on morphology and growth curve analyses were performed regularly, and no phenotype changes were observed throughout the duration of this study. Short tandem repeat (STR) profiling of the cell lines revealed there is no match with publicly available profiles of other cell lines, and that the cell lines are unique and are not cross-contaminated or misidentified. All methods were carried out in accordance with the approved guidelines of our institution.

### 2.2. Tumor-Spheres Generation and Migration Analysis

For tumor-spheres generation, cells were cultured in a spheroid formation media (growth culture medium supplemented with 0.2% methylcellulose) in non-adhesive convex environment for 12 h at 37 °C and 5% CO_2_ using the hanging drop cell protocol. Tumor-spheres were mixed with collagen matrix (2.5 mg/mL) and incubated for 30 min at 37 °C prior to microscopic analysis. For co-culture experiments, fibroblasts were labeled with the fluorescent dye CellTracker™ Green CMFDA (5-chloromethylfluorescein diacetate; Thermo Fisher) following the manufacturer’s instructions. Then, equal amounts of cells were used for tumor-spheres generation (see Figure 3A). For quantification of collective cell migration, the spheroid areas were measured at each time point using the Zen software and the tool contour (spline) to define the Spheroid Cross-Sectional Area (SCSA) and calculate its variation over time. To quantitatively analyze the variations of the contour length of the spheroids, we used the previously described shape factor α, which is the ratio of the SCSA over the contour length of the interface normalized by half the instantaneous radius R [17].

### 2.3. Immunohistochemistry and Immunofluorescence

Formalin-fixed, paraffin-embedded tissues were cut into 3-μm sections and mounted on poly-L-lysine-coated slides (DakoCytomation). Antigen retrieval was performed by heating for 20 min in a pressure cooker with an Envision™ FLEX target retrieval solution with a pH of 9. Tissue slides were incubated for 1 h with the following primary antibodies: mouse IgG anti-E-cadherin antibody (Becton Dickinson Transduction Laboratories, Erembodegem, Belgium) at a 1:500 dilution, mouse IgG anti-N-cadherin (Dako, Agilent Technologies) at a 1:200 dilution, mouse anti-cytokeratin (Dako, Agilent Technologies) at a 1:50 dilution, rabbit anti-vimentin (Abcam) at a 1:200 dilution and mouse anti-58K Golgi protein (Abcam) at a 1:50 dilution. For immunohistochemistry and for each antibody, all the slides were stained simultaneously in an automated horizontal slide-processing system (Dako Autostainer Plus). Negative controls with either an omission of the primary antibody or with a normal mouse IgG (Santa Cruz Biotechnology, Inc.) in the primary incubation were also included. The slides were digitalized on a Leica SCN400F scanner and images were visualized and extracted with the SlidePath Gateway LAN software. Images were analyzed randomly by three of the authors without knowledge of the clinicopathological data. Two of the tissue samples were also analyzed by double labeling immunofluorescence with anti-E-cadherin and anti-N-cadherin. Anti-rabbit IgG Alexa Fluor 488 and anti-mouse IgG Alexa Fluor 555 were used as secondary antibodies at 1:500 dilutions for 1 h. Immunofluorescence stainings were analyzed on a Zeiss AxioObserver Z1 microscope (Carl Zeiss, Germany) with a Plan-Apochromat 40X/1.3 (NA = 1.3, working distance = 0.21mm) or Plan-Apochromat 63X/1.4 (NA = 1.4, working distance = 0.19mm) oil lens objective, a camera (AxioCam MRm; Carl Zeiss) and an Apotome (ApoTome 2; Carl Zeiss).

### 2.4. Time-Lapse Microscopy

Time-lapse microscopy imaging was performed on a Zeiss AxioObserver Z1 microscope (Carl Zeiss, Germany) with a Plan-Apochromat 40×/1.3 (NA = 1.3, working distance = 0.21 mm) or Plan-Apochromat 63X/1.4 (NA = 1.4, working distance = 0.19mm) oil lens objective, a camera (AxioCam MRm; Carl Zeiss) and an Apotome (ApoTome 2; Carl Zeiss). Z-stack images were taken with the AxioVision module Z-stack (Zeiss).

### 2.5. Statistical Analysis

The two-tailed independent Student t-test was used to compare the variables between two groups. An ANOVA test (for more than two groups) was performed to compare the mean among groups. All data were derived from independent experiments. The level of statistical significance was set at 0.05 for all tests. In silico analysis of published datasets was performed by using the CANCERTOOL software and the Basic Analyses section, which generated survival curves using the Kaplan–Meier method [18]. A Mantel-Cox test was performed to compare the differences between survival curves, while a Cox proportional hazards regression model was performed to calculate the Hazard Ratio (HR) between groups. The Log2-normalized gene expression represents the fluorescence intensity values for the microarray data or sequencing read values obtained after gene quantification with RSEM, with normalization using Upper Quartile in case of RNAseq.

## 3. Results

### 3.1. Phenotypic Heterogeneity and Cell-To-Cell Interactions of Human SCC-Derived Cells

**Figure 1:**
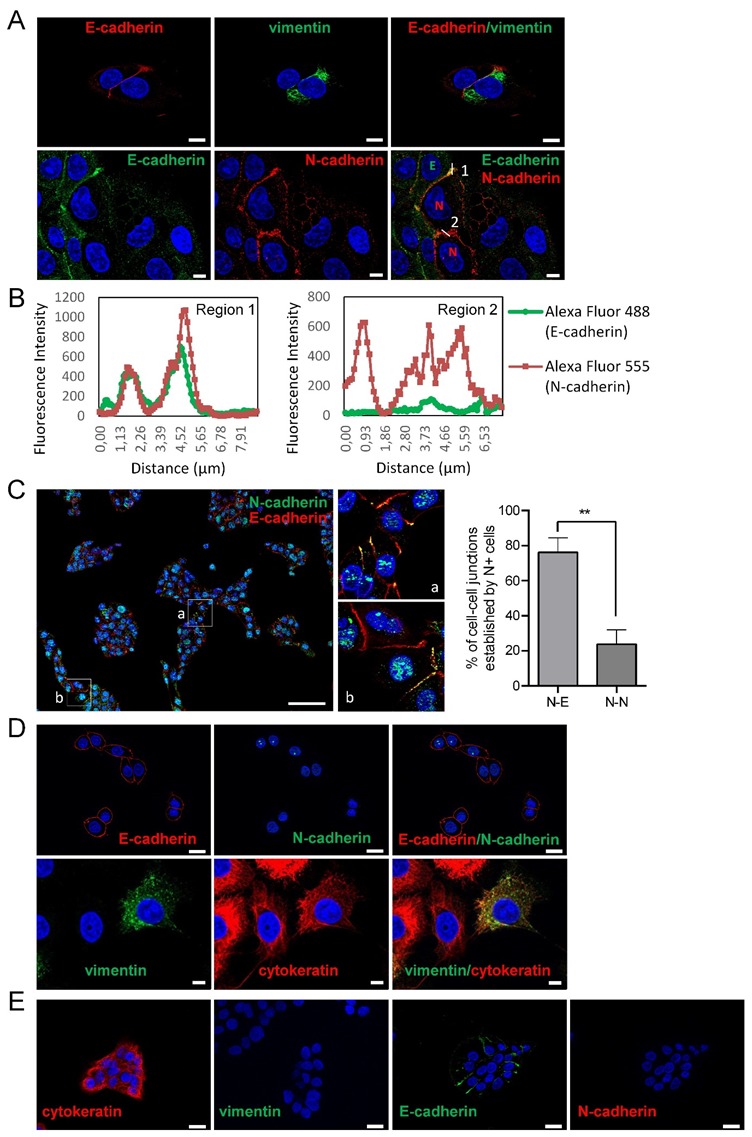
Expression of epithelial and mesenchymal markers in SCC cell lines. Representative immunofluorescences of UT-SCC-40 (**A**,**C**), UT-SCC-42B (**D**) and UT-SCC-38 (**E**) with antibodies against E-cadherin and cytokeratin, as epithelial cell markers, and N-cadherin and vimentin, as mesenchymal cell markers. All pictures (except the two images at the left site of the (**E**) panel) represent double immunofluorescence labeling with the indicated antibodies. Cell nuclei were stained with 4′,6-diamidino-2-phenylindole (DAPI) (blue). (**B**) Graphics show the fluorescence intensity profiles of the regions of interest, 1 and 2, indicated with a white line in panel (**A**). Note the co-localization of E-cadherin and N-cadherin at the membrane at the junctions between the E+ and N+ cells (represented by green E and red N in the picture at the right bottom) and the absence of E-cadherin labeling at the junction between the two N+ positive cells. The image in (**C**) is a representative tiled image of cell culture showing that N+ cells do not preferentially interact with N+ cells. Quantification of the percentage of cell–cell junctions (N+-N+ or N+-E+ junctions) established by N+ cells is shown at the right of panel C (113 total contacts counted in 5 independent experiments). Higher magnifications of the a and b regions are showed in the right images. Scale bars: 20 µm (panels **A**,**D**,**E**) and 100 µm (panel **C**). ** *p* < 0.005.

Here, we have sought to analyze the mode of collective cell migration of head and neck SCC-derived cell lines according to their epithelial or mesenchymal phenotype. To this end, we have used three cell lines containing phenotypically homogeneous (UT-SCC-38 cells) or heterogeneous (UT-SCC-40 and UT-SCC-42B cells) cell populations. As previously reported [16], the UT-SCC-40 cell line contains cells with epithelial phenotypes expressing cytokeratin/E-cadherin but not vimentin/N-cadherin (CK+VIM-/E+N-) and cells with mesenchymal phenotypes expressing vimentin/N-cadherin but not cytokeratin/E-cadherin (VIM+CK-/N+E-) in an approximate 3:1 ratio (Figure 1A). Analysis of the cell-to-cell interactions in this cell line revealed the presence of contacts between cells of the same phenotype and also of a distinct phenotype. Indeed, homotypic E+/E+ or N+/N+ junctions as well as heterotypic N+/E+ junctions were detected by immunofluorescence (Figure 1A,C). As shown in Figure 1C, UT-SCC-40 cells tend to growth as cell clusters that contain both N+ and E+ cells. Within those clusters, the junctions between adjacent N+ cells were found to be even less abundant than contacts established between N+ cell and E+ cells (23.75% ± 8% versus 76.24% ± 8.22%; *p* = 0.002), thus suggesting that UT-SCC-40 cells are prone to have promiscuous interactions between them.

The co-localization of the N-cadherin signals with E-cadherin at the adhered cell membranes suggests the presence of heterotypic E-cadherin and N-cadherin trans interactions. Similarly to UT-SCC-40 cells, the UT-SCC-42B cell line also contain a mixed population of cells but, in this case, cells are either of epithelial (CK+VIM-/E+N-) or of a hybrid epithelial/mesenchymal phenotype (CK+VIM+/E+N-) in an approximate 1:1 ratio (Figure 1C) [16]. Contrary to UT-SCC-40 and UT-SCC-42B cells, the UT-SCC-38 cell line contains a unique and homogeneous population of epithelial CK+VIM-/E+N- cells (Figure 1D) [16].

### 3.2. Human SCC-Derived Cells, but not Fibroblasts, Display a Collective Mode of Invasion

**Figure 2:**
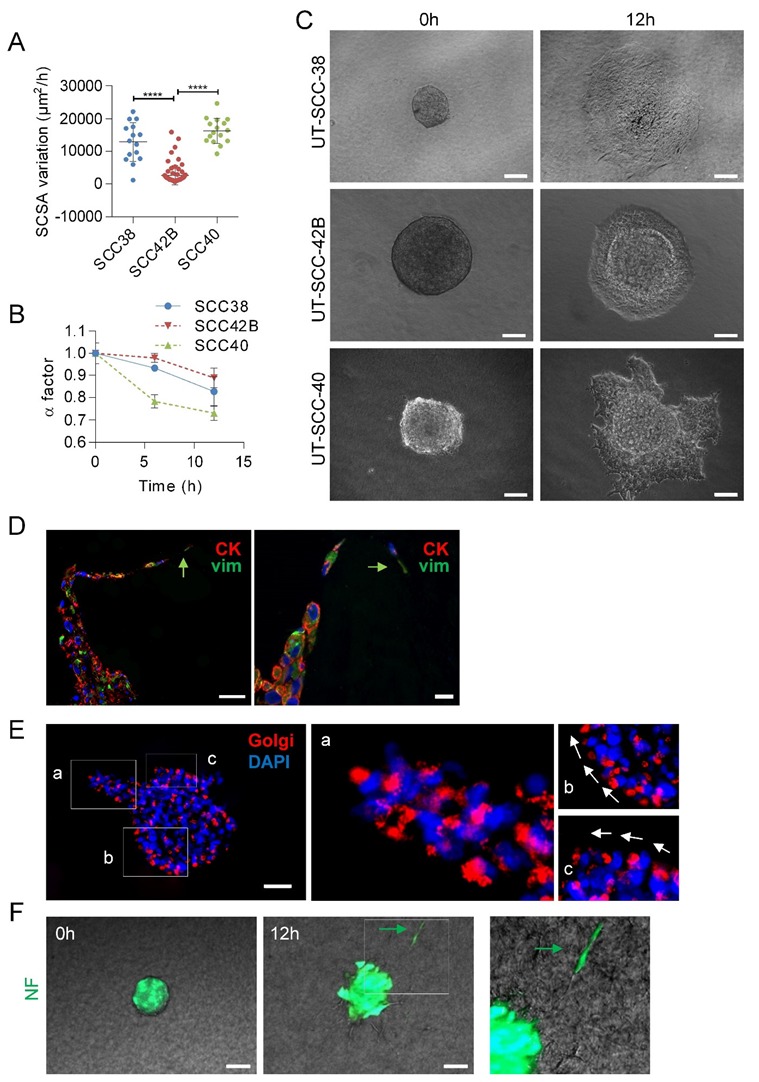
Collective migration of different SCC cell lines showing the driver role of mesenchymal cancer cells over epithelial cells. (**A**) Variations over time of the spheroid cross sectional areas (SCSA) were measured for 12 h in spheroids of the indicated cell lines to calculate the velocity of the migration. Mean and standard deviation values were calculated from 15, 68 and 16 individual spheroids in 3 different experiments for UT-SCC-38, UT-SCC-42B and UT-SCC-40 cells, respectively. **** indicates *p* < 0.0001. (**B**) Variation over time of the α factor for the indicated cell lines. Data are presented as mean value ± standard deviation from 3 experiments and 10–20 spheroids analyzed in each. (**C**) Representative images of spheroids assembled with the indicated SCC cells at time 0 and after 12 h of incubation. (**D**,**E**) Representative images of UT-SCC-40 cell spheroids after 24 h of migration immunostained with anti-cytokeratin (red) plus anti-vimentin (green) antibodies (**D**) or anti-58K Golgi protein antibody (**E**). Green arrows denote vimentin positive cells acting as leader of the finger-like cell tracks (**D**). Higher magnification of the areas (a–c) outlined in the left image of panel E are shown at the right to highlight the position of the Golgi apparatus ahead of the nuclei of SCC cells coordinately polarized. White arrows denote the SCC cell’s movement direction. (**F**) Type of migration of non-tumoral fibroblasts (NF) assembled into cell spheroids. Representative images from time-lapse movies of cell spheroids assembled with NFs embedded into a collagen matrix. Cells were labeled with CellTracker green CMFDA before spheroids assembly. Green arrow points to a NF-cell escaping from the cell spheroid. Scale bars: 100 µm (panels **C**,**F**), 50 µm (left picture in panel **D** and panel **E**) and 20 µm (right picture in panel **D**).

First, we checked that the different SCC cell lines recapitulate, under in vitro conditions, the collective mode of invasion. Video microscopy experiments of SCC cell spheroids revealed that the clusters of cells migrated into the collagen matrix in a coordinated fashion by maintaining cell-to-cell contacts without any cell detachment from the tumor-spheroid (Videos S1–S3). Next, we compared the variations over time of the invasion rate of the three SCC-derived cell lines. As shown in Figure 2A, UT-SCC-40 and UT-SCC-38 cells migrated faster than UT-SCC-42B cells, which is in accordance with our previous observations in 2D systems [19]. The borders of the tumor-spheroids adopted an irregular shape over time due to the extension of cellular protrusions or the presence of collective invasion of finger-like cell strands into the three-dimensional extracellular matrix. Thus, we quantitatively analyzed the variations of the length of the contour of the tumor-spheroids by using a previously described shape factor (α) (see Methods section). This parameter, which range from 0 (very irregular interface) to 1 (perfect circle), slightly decreased from 1 ± 0.009 to 0.82 ± 0.06 or 0.88 ± 0.043 in a 12 h period in UT-SCC-38 and UT-SCC-42B cells, respectively, indicating that, despite the presence of some lamellipodia, the movement of cells was rather isotropic without severe fingering activity. By contrast, the pronounced irregular shape of the tumor-spheroids was observed in UT-SCC-40 cells with a decrease of α factor from 1 ± 0.04 to 0.7 ± 0.03 in 12 h of invasion due to the presence of finger-like cell strands protruding out of the tumor-spheroids (Figure 2B,C). This suggests the presence of a functionally heterogeneous UT-SCC-40 cell population containing fractions of cells with higher invasive behavior than others.

Given that the UT-SCC-40 cell line contains a heterogeneous population of CK+VIM-/E+N- and VIM+CK-/N+E- cells, we sought to determine what phenotype had the cells that took the leader positions of the cell strands in UT-SCC-40 spheroids by using immunocytochemical analysis of CK and VIM after 20 h of migration of the cell spheroids. Analysis of the location of the Golgi apparatus was used as a marker of cell polarity (Figure 2E). The data revealed that cells at the finger-like strands were collectively polarized and that the leader cells expressed VIM whereas cells at the rear positions expressed CK (Figure 2D,E). This suggests that mesenchyme-like UT-SCC-40 cells forced the migration of the epithelial-like SCC cell by acting as leader tractor cells of the invasive cell strands.

Contrary to SCC-derived tumor cells, non-tumoral fibroblasts (NF) and CAFs derived from two independent human head and neck SCCs (CAF1 and CAF3, see Figure S1) grouped in spheroids, and mostly did not invade the collagen matrix (Video 4). Occasionally, a small fraction of NFs was found to penetrate into the collagen matrix, but these cells detached from neighbor cells and migrated as individual units, not as a cohesive group of cells (Figure 2F). Thus, effective collective cell invasion seems to be a specific hallmark of tumor SCC cells.

### 3.3. CAFs as Leaders of Collective Migration of SCC Cells

**Figure 3:**
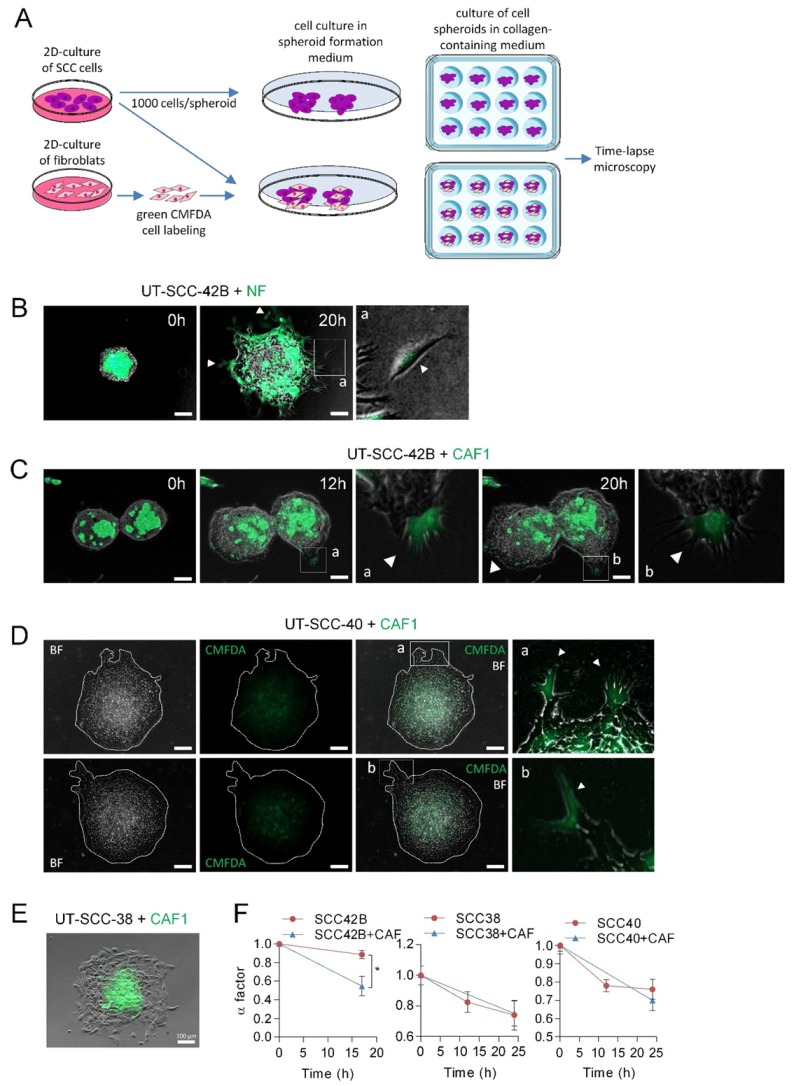
Leadership of CAFs in the invasive fronts of CAF + SCC mixed spheroids. (**A**) A scheme showing the protocol for assembly of SCC or SCC + CAF spheroids. Hanging drop cell cultures were done by using 1000 cells/spheroid, either SCC cells or CMFDA-labeled fibroblasts plus SCC cells (500 cells each), to form cell spheroids that were subsequently embedded in a collagen medium and time-lapse recorded. (**B**–**E**) Representative images of spheroids assembled with mixed populations of SCC cells and NF or CAF1 at the indicated times of incubation. Fibroblast-cell lines were labeled with green CMFDA to allow their tracking over time. Pictures a and b (panels **B**–**D**) are magnified images of the indicated insets to highlight the presence of CMFDA-labeled fibroblasts. White arrows point to NF cells slipping away from the spheroid (panel B) and CAFs leading the invasive finger-like cell tracks in panels (**C**,**D**). Pictures in panel D are representative bright field (BF) and fluorescence (CMFDA) images of two mixed SCC + CAF spheroids that were allowed to migrate for 24 h. Dashed white lines show the limits of the spheroid. Scale bars: 100 µm. (**F**) Variation over time of the α factor for the indicated cell spheroids. Data are presented as the mean value ± standard deviation from 3 experiments and 10–20 spheroids analyzed in each. * indicates *p* < 0.05.

Next, we tested whether CAFs could act as leaders in the SCC-cell spheroids such as it was described in other types of cancers [12,14,20]. To this end, mixed spheroids composed by equal amounts of UT-SCC-42B cells (which did not form, by themselves, invasive finger-like cell tracks) and NFs or CAFs were assembled as shown in Figure 3A. To identify NFs or CAFs, these cells were labeled with green CMFDA. Under these conditions, NFs migrated out of the spheroids as single cells such that collective cell migration of NF+SCC cells did not occur (Figure 3B). By contrast, the presence of CAFs in the spheroids induced the formation of protrusions at the periphery of the spheroids, which were led by one CAF and followed by UT-SCC-42B cells (Figure 3C and Supplementary Videos S5 and S6). Accordingly, the α factor decreased from 0.88 ± 0.04 to 0.54 ± 0.10 in a 17 h period, 49% more than observed in spheroids composed only by UT-SCC-42B cells (Figure 3F), and most (about 90%) of the finger-like protrusions were led by a CAF.

In accordance to the above data, CAFs also acted as leaders of a small fraction of invasive cell tracks developed in CAFs + UT-SCC-40 mixed spheroids (Figure 3D). In this case, however, the number of finger-like cell tracks did not significantly increase since differences in the α factor between UT-SCC-40 and UT-SCC-40+CAF spheroids were not observed (Figure 3F). In contrast to the invasive behavior observed in mixed CAF+UT-SCC-42B and CAF+UT-SCC-40 spheroids, CAFs did not promote the invasive activity of UT-SCC-38 cells nor took the leader position on the invasive front (Figure 3E,F). In fact, in this case, CAFs remained at the center of the spheroid. Thus, the interaction between the CAFs and SCC cells may depend on a tumor’s intrinsic properties.

**Figure 4:**
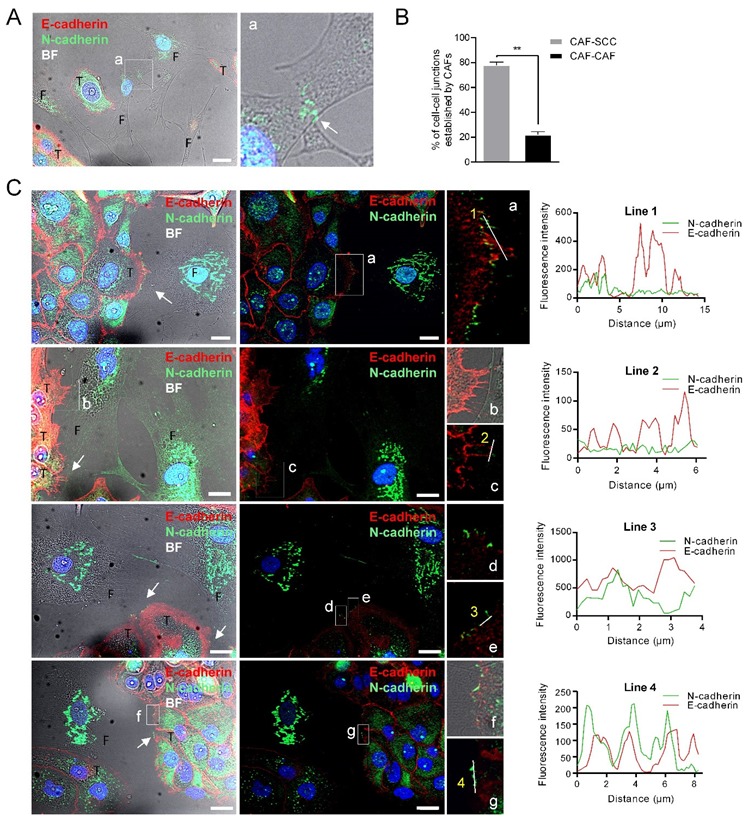
N-cadherin does not co-localize with E-cadherin at the membrane contacts established between CAFs and E+ SCC cells. Representative bright-field and immunofluorescences of UT-SCC-42B plus CAF3 co-cultures with anti E-cadherin (red) and N-cadherin (green) antibodies. Panel (**A**) shows the interaction between two CAFs; a higher magnification of the region (a) denoted by a white square is shown at the right picture to highlight the N+-cadherin mediated interaction. Panel (**B**) shows the percentage of cell–cell junctions (CAF-SCC+ or CAF-CAF) established by CAFs (376 total contacts counted in 3 independent experiments). Panel (**C**) shows four representative examples of the interactions between CAFs and SCC cells. Higher magnifications of the cell-to-cell contacts are shown in the far-right images (a–g). White arrows point to cell-to-cell contacts. Graphics show the fluorescence intensity profiles of the regions of interest indicated with white lines in the fluorescence pictures. T, tumor cell; F, CAF3. Scale bars: 20 µm.

To determine whether heterophilic E-cadherin/N-cadherin trans interactions were formed between CAFs and SCCs as previously reported [14], we co-cultured UT-SCC-42B cells, which express E-cadherin but not N-cadherin, with CAF3 cells. We found that junctions between CAFs and SCC cells were more frequent than CAF–CAF cell junctions (79% ± 1.8% vs. 20.38% ± 1.8%; *p* = 0.0013) (Figure 4B). Cell-to-cell contacts between SCC cells contained E-cadherin whereas junctions between CAF3 cells had N-cadherin, as expected (Figure 4A). Contacts between SCCs and CAFs were readily observed and both E-cadherin and N-cadherin proteins were detected at the SCC-42B-CAF3 cell junctions (Figure 4C). However, these two cell adhesion proteins did not co-localize in contrast to what it had been observed in the UT-SCC-40 cell line. Thus, N-cadherin present in CAFs likely interact in trans with cadherin family member/s other than E-cadherin or with other membrane proteins, thus promoting tumor cell dragging actions.

### 3.4. Leader-Like Positions of Collective Migration of N-Cadherin-Expressing Tumor Cells and CAFs in Human SCC Tissues

**Figure 5:**
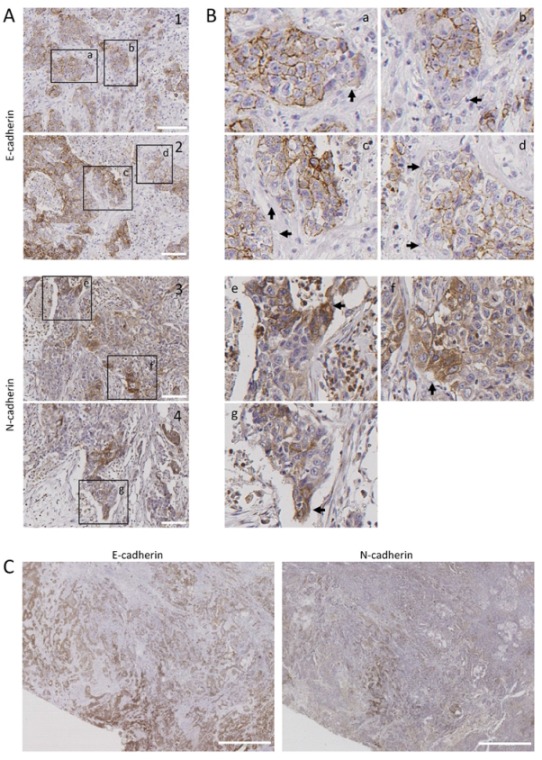
Distribution of E-cadherin and N-cadherin cells in human head and neck SCCs. Representative images from E-cadherin and N-cadherin immunohistochemical stainings of surgically treated head and neck SCC. Panels (**A**,**B**) show representative tumor sections showing E-cadherin (1, 2) or N-cadherin (3, 4) immunostainings. Higher magnifications of the areas labeled in panel A are shown in panel B. Black arrows point to tumor cells with low E-cadherin (a–d) or high N-cadherin immunostainings (e–g). (**C**) Serial sections of a tumor tissue showing immunostaining of N-cadherin in tumor areas negatively/weakly stained with anti E-cadherin antibody. Scale bars 100 µm (**A**) and 2000 µm (**C**).

**Figure 6:**
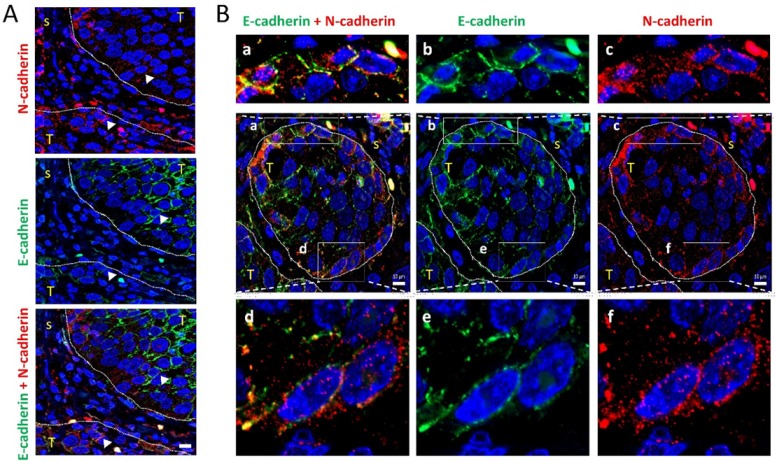
Colocalization of E-cadherin and N-cadherin in human head and neck SCCs. Double immunofluorescence labeling of two head and neck SCC tissue (**A**,**B**) for E-cadherin (green) and N-cadherin (red). Higher magnifications of the areas outlined in the pictures are shown at the upper and bottom parts of panel B to highlight the co-localization of E- and N-cadherin at the cell periphery. Dashed white lines denote the limits between tumor (T) and stromal (s) areas of the tissue. Scale bars: 10 µm.

To assess the clinical relevance of our findings, we inspected tumor tissues for the presence of tumor N-cadherin-expressing cells to analyze their tissue distribution. Immunohistochemical analysis of N-cadherin in 22 human patient-derived head and neck SCC tissues revealed that 23% of them contained tumor cells with positive immunostaining in cell membranes. These cancer cells were heterogeneously distributed. They were mostly located at discrete positions at the periphery of tumor nests (Figure 5A,B). As expected, loss of E-cadherin immunostaining was detected also at poles of tumor nests. More importantly, regions of tumor tissues with low E-cadherin immunostaining had N-cadherin-positive cells (Figure 5C), suggesting the existence of in vivo cadherin switching. Double immunofluorescence was performed in two N-cadherin-positive tissues to simultaneously detect E- and N-cadherin. This analysis showed that the invasive tumor nests contained E+N- cells in the inner region and N+E- cells in the leading edges (Figure 6A). In addition, co-localization of N-cadherin and E-cadherin was detected in some tumor cells of the periphery of the tumor nests (Figure 6A,B), suggesting the presence of heterophilic E-cadherin–N-cadherin cell-to-cell interactions.

**Figure 7:**
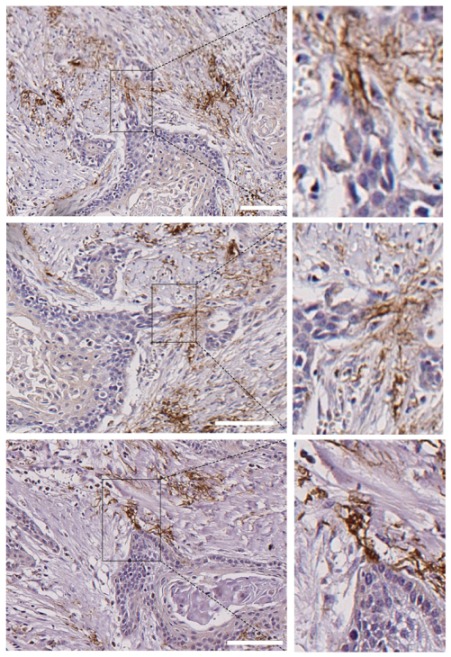
Presence of N-cadherin stromal cells in contact with the tips of tumor nests in human head and neck SCCs. Submitted as separate file to keep proper resolutions of images. Three different areas of a single head and neck SCC that contain tumor nests with invasive N- tumor cells that are connected with stromal N+ cells at the tips of the invasive cell tracks. Higher magnifications of the areas outlined in the right pictures are shown in the left pictures. Scale bars: 100 µm (upper and bottom images) and 50 µm (image at the middle).

Most tumor tissues showed a very weak N-cadherin staining at the stromal cells. However, 2 out of the 22 tissues (9%) contained a remarkable strong positivity at the stromal cells and these were found at tips of the invasive edges of the tumor cell nests (Figure 7).

### 3.5. Clinical Outcome of Patients with Head and Neck SCC Containing “Leader” N-Cadherin-Expressing Cells

To evaluate the clinical significance of N-cadherin expression, we examined the associations between N-cadherin protein expression and clinical data in the 22 patients with head and neck SCCs. N+ tumors were defined as those containing N-cadherin immunopositive tumor or stromal cells. N- tumors were those with N- tumor cells that lack N+ CAFs.

**Figure 8:**
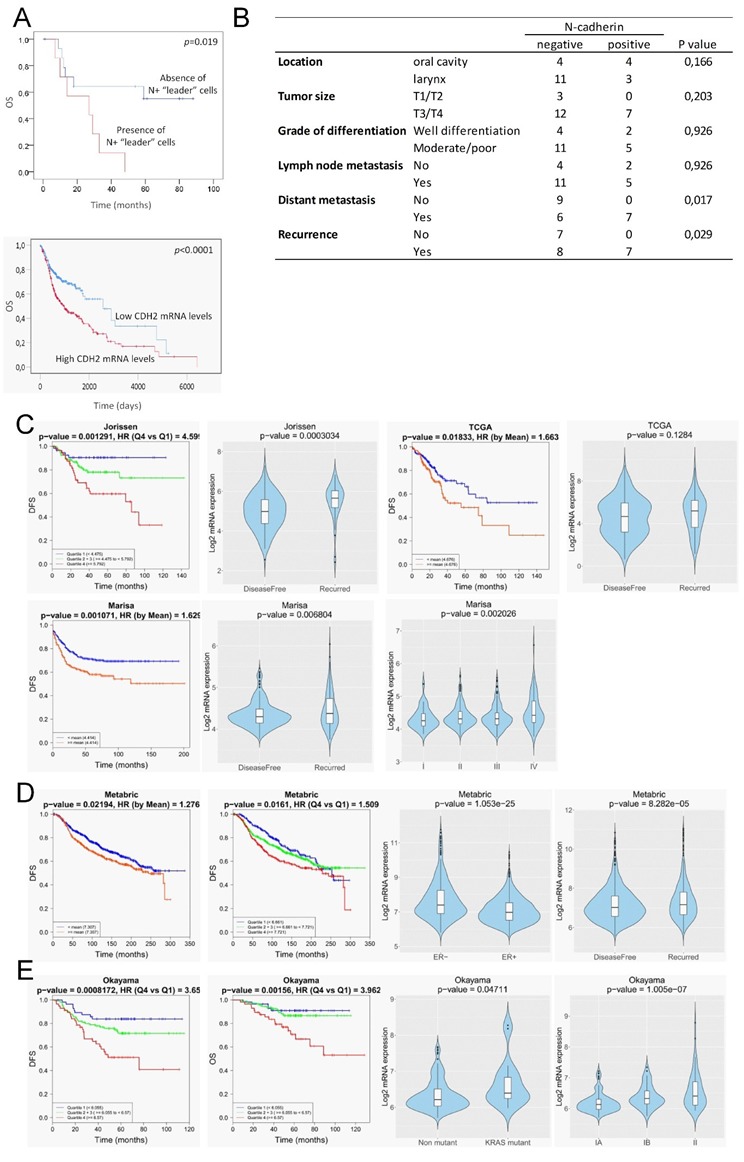
Clinical outcome associated with the N-cadherin expression in head and neck SCCs and other epithelial cancer types. (**A**, up) Kaplan–Meier estimate of overall survival (OS) among patients with head and neck SCCs that are classified according to the presence or absence of N-cadherin-expressing “leader” cells (either cancer cells or CAFs). (**A**, down) Kaplan–Meier estimate of overall survival (OS) among TCGA patients with head and neck SCCs that are classified according to the levels of CDH2-mRNA: high (above the median value of CDH2 mRNA levels) and low (below the median value of CDH2-mRNA levels). (**B**) Correlations between the presence of N+ “leader” cells in head and neck SCC and clinical variables. (**C**–**E**) In silico N-cadherin mRNA expression comparative analyses between different groups of patients with colorectal (**C**), breast (**D**) and lung (**E**) cancer using published datasets. Graphics include Kaplan–Meier curves representing the disease-free survival (DFS) or overall survival (OS) of patient groups selected according to the quartile expression of N-cadherin in the datasets designated as indicated above each graphic. Quartile color code: Q1 (Blue), Q2 plus Q3 (Green) and Q4 (Red). Violin plots depict the expression of the N-cadherin among cancer specimens of the indicated group of tumors in the different datasets. Pathological stages are indicated as IA, IB, II, III, IV. ER- and ER+ refers to estrogen receptor negative and positive breast cancer specimens, respectively. Non-mutant and KRAS mutant refer to lung adenocarcinoma specimens with the wild type and mutated KRAS, respectively.

We found that N-cadherin positivity was significantly associated with distant metastasis and tumor recurrence; 100% of patients with N+ tumors had developed metastasis or disease recurrence as compared with N- tumors that developed distant metastasis or had disease recurrence in 40% and 53% of cases, respectively (*p* = 0.017 and *p* = 0.029, respectively; Figure 8B). In addition, the presence of N+ “leader” cells in tumors was significantly associated with low overall survival as compared with tumors lacking these cells (*p* = 0.019) (Figure 8A). No significant associations were found with other histopathological data or with lymph node metastasis. Independent analysis of the impact of the expression levels of the N-cadherin (CDH2) in head and neck SCCs using TCGA datasets also revealed that high CDH2-mRNA levels significantly correlates with low overall survival (Figure 8A).

### 3.6. In Silico Analysis of N-Cadherin Aberrant Expression and Clinical Outcome in Patients with Breast, Colorectal and Lung Cancer

Our findings that strong N-cadherin protein expression occurs only in tissues containing “leader” cells, either cancer cells or CAFs, led us to postulate that high N-cadherin mRNA levels could be a surrogate marker of mesenchymal cell-led collective invasion of carcinoma cells. Because collective cell migration is of paramount importance in the metastatic behavior of other types of epithelial cancer, we sought to determine whether our findings in head and neck SCC could have a broader significant meaning. To this end, we interrogated well-annotated datasets with rich clinical annotations to estimate whether increased N-cadherin mRNA levels is associated to disease progression in other prevalent tumors such as colorectal, breast and lung cancer. This analysis was performed by using the basic and survival analysis provided by the CANCERTOOL software that provides rapid and comprehensive visualization of gene expression data for the gene(s) of interest in several well-annotated cancer datasets and the TCGA datasets [18]. Figure 8C–E shows the most significant findings that were obtained. Three out of six independent datasets on colorectal cancer [21,22,23,24,25] revealed that N-cadherin mRNA levels were significantly lower in cancer tissues from patients that remained disease-free than in those that exhibited recurrence. Moreover, N-cadherin mRNA levels increased as the pathological stage of the tumors increased. Disease-free survival of patients was significantly lower when the tumor overexpressed N-cadherin as compared with low-expressing tumors. Cohort sizes of those datasets were 290 (Jorissen) [25], 585 (Marisa) [22] and 374 (TCGA) patients (Figure 8C). With regard to breast cancer datasets, one of the six available studies [26,27,28,29,30], the one containing the largest cohort size (METABRIC, *n* = 1980) [29], revealed that estrogen receptor negative tumors had a significantly higher N-cadherin mRNA than estrogen receptor positive tumors. As observed in colorectal cancer, N-cadherin overexpression positively correlated with disease recurrence. Moreover, disease-free survival of patients was significantly lower when the tumor overexpressed N-cadherin as compared with low-expressing tumors (Figure 8D). In the case of lung adenocarcinomas, only one of the five independent datasets [31,32,33,34] that were available yielded significant findings (Okayama, cohort size: 246 patients) [33]. In this study, high levels of N-cadherin mRNA were significantly associated with a Kras mutation, a more advanced disease and a lower disease-free and overall survival than that observed in patients with low N-cadherin expression (Figure 8E). In contrast to the data in N-cadherin expression, the analysis of E-cadherin mRNA levels in the same datasets revealed that loss of E-cadherin was not associated with progressive disease in any of the analyzed datasets (Figure S2).

## 4. Discussion

This work reveals the distinct leader and follower roles of mesenchymal and epithelial cancer cells and CAFs in the plasticity of collective cancer migration of SCC cells. Our in vitro model system of collective cell migration cells has revealed for the first time that, when epithelial E+/CK+ and mesenchymal N+/VIM + SCC cells cohabit, N+VIM+ cells act as leaders of the collective cell migration dragging E+CK+ cells with them. Similarly, CAFs also drive the collective cell movements of E+CK+ SCC cells in agreement with previous reports [12,14,20]. More importantly, we show for the first time that these results are consistent with the topographic distribution of N+ cancer cells and CAFs observed in head and neck SCC tissues that had metastatic behavior.

Comparing to single cell migration, less effort has been directed towards understanding how tumor cell heterogeneity and EMT contributes to collective cell migration. Individual cancer cell migration is accepted to occur by the loss of E-cadherin, which results in weakened cell junctions followed by cell detachment and the onset of a single-cell mode of migration [4,5,6]. Nevertheless, the decrease in E-cadherin is often associated with an increase in N-cadherin in a process called cadherin switching [7]. Because cadherin binding in trans, between adjoining cells, preferentially involve identical cadherin molecules [35], cadherin switching implies that the N+ cancer cells preferentially interact with the surrounding stromal fibroblasts, which normally express N-cadherin, rather than with E+ cancer cells, thereby escaping from the tumor nests. Here, we provide novel data showing that heterotypic E-cadherin/N-cadherin contacts can be established between the epithelial and the mesenchymal cancer cells. This enable N+ cancer cells to remain adhered to E+ cancer cells whom they drag towards the surrounding stroma forming a cohesive tumor invasive nest containing E+ and N+ cancer cells. Thus, besides the well-established role of E-cadherin-based adherent junctions as the dominant mediator of collective cell interactions [11], N+ cancer cells also contribute to the plasticity of collective cell invasion by acting as leaders of the cooperative cell movements. Previous reports have shown that cancer cells adhered via N-cadherin–N-cadherin interactions can also migrate collectively but, in contrast to our data, promotion of a “leader–follower” mode of collective cell invasion was not observed in co-cultures of E+ and N+ cells [36]. Importantly, we reveal here that N+ and E+ cancer cells are asymmetrically distributed in head and neck SCC tumor tissues, with N+ cells at the invasive fronts of tumor nests, matching with areas of E- cancer cells, and E+ cells behind them. This is suggestive of a front–rear asymmetry, which is a feature of all migrating collectives. This supports our in vitro findings and the involvement of N+ cancer cells in the plasticity of collective cell migration. This is the first study that show evidences that the leadership function of mesenchymal cells in collective cell movements may operate in vivo as well. It remains to be established whether this behavior is cancer-type specific.

It has been established earlier that CAFs may also be involved in collective cancer cell migration by remodeling the extracellular matrix and creating tracks for cancer cells [12,13,20]. More recent data have demonstrated that CAFs can also actively drive collective migration of cancer cells via the establishment of heterophilic E-cadherin–N-cadherin adherens junctions [13]. We have confirmed the leadership role of CAFs in collective cell invasion of SCC cells in vitro. The novelty of our study is that “leader” CAFs have been also detected in head and neck SCC tumor tissues. A small percentage of tumor tissues (about 9%) harbored strongly immunostained N+ stromal cells whereas this immunostaining was rather weak in the rest of cases. Importantly, the strongly stained N+ stromal cells were detected at the tips of the most invasive fronts of tumor nests in close connection with cancer cells as if they were dragging them. These observations support the role of CAFs in the plasticity of collective migration. Interestingly enough, the tumors with that phenotype did not contain N+ cancer cells, suggesting that CAFs supplant N+ cancer cells in the promotion of metastasis. Why this behavior of CAFs is adopted in some tumors but not in others is an important issue that remain to be explored. Our in vitro data revealed that the same CAF-cell lines are able to guide collective migration of some cells (UT-SCC-40 and UT-SCC-42B) but not of others (UT-SCC-38) cells. This suggests that signaling directed from SCC cells towards CAFs have a dominant role in the promotion of the leadership phenotype of CAFs, over those directed from CAFs to SCC cells. In contrast to a previous report [13], when E+ cancer cells and CAFs were co-cultured, E-cadherin did not co-localize with N-cadherin at the joining plasma membranes between SCC cells and CAFs. Even more, in tumor tissues, the cancer cells in the invasive fronts “led” by CAFs were E- and also N-, thus implying that molecules other than E-cadherin or N-cadherin contact with N-cadherin at the CAF membranes. Cadherin switching is not limited to E-to-N cadherin in epithelial cells and, therefore, other ectopically expressed cadherins may play a role in SCC–CAF interaction [37].

Aberrant N-cadherin expression has a clear role in tumour progression in epithelial cancer cells and has been associated with poor overall patient survival in diverse types of cancers [38]. Accordingly, we have found that the presence of leader mesenchymal cells, either N+ cancer cells or CAFs, is significantly associated with distant metastasis and low overall survival in patients with head and neck SCC. Data derived from in silico analysis of N-cadherin mRNA in other epithelial cancers reinforced the notion that the cooperation of mesenchymal cells with epithelial SCC cells favors distant metastasis. High N-cadherin mRNA levels in tissues of breast, lung and colorectal cancer is associated with disease recurrence and low survival of patients. These findings, however, were not replicated when analyzing decreased E-cadherin expression in those tumors. Thus, cadherin switching rather than simply E-cadherin loss has a dominant effect as driver of metastasis in epithelial cancers by enabling E+ cancer cells to reach the metastatic sites, among other possibilities. Collectively, our data clearly show the importance of cooperation among the heterogeneous cell subpopulations within a tumor and their complex interactions that control cancer cell invasion and affect the clinical outcome of cancer patients. These findings provide insights into how to better target pathological cell migration, thereby improving current strategies for suppressing tumor cell invasion and the onset of metastasis.

## Supplementary Materials

The following are available online at https://www.mdpi.com/2073-4409/9/3/731/s1, Figure S1. Phenotypic characterization of fibroblasts. Figure S2: Clinical outcome associated with the E-cadherin expression in head and neck SCCs and other epithelial cancer types.
