# Supplementary material for: The Leader Position of Mesenchymal Cells Expressing N-Cadherin in the Collective Migration of Epithelial Cancer
Source: Cells. 2020 Mar 16;9(3):731. doi: 10.3390/cells9030731 (PMC7140612; doi:10.3390/cells9030731)
Supplement: Supplementary file 1 [file cells-09-00731-s001.zip › cells-721204-SI/cells-721204supp revised.pdf]

Supplementary Material
Leader position of mesenchymal cells-expressing N-cadherin- in collective migration of epithelial cancer

Inés Saénz-de-Santa-María, Lucía Celada and María- Dolores Chiara

FIGURE S1

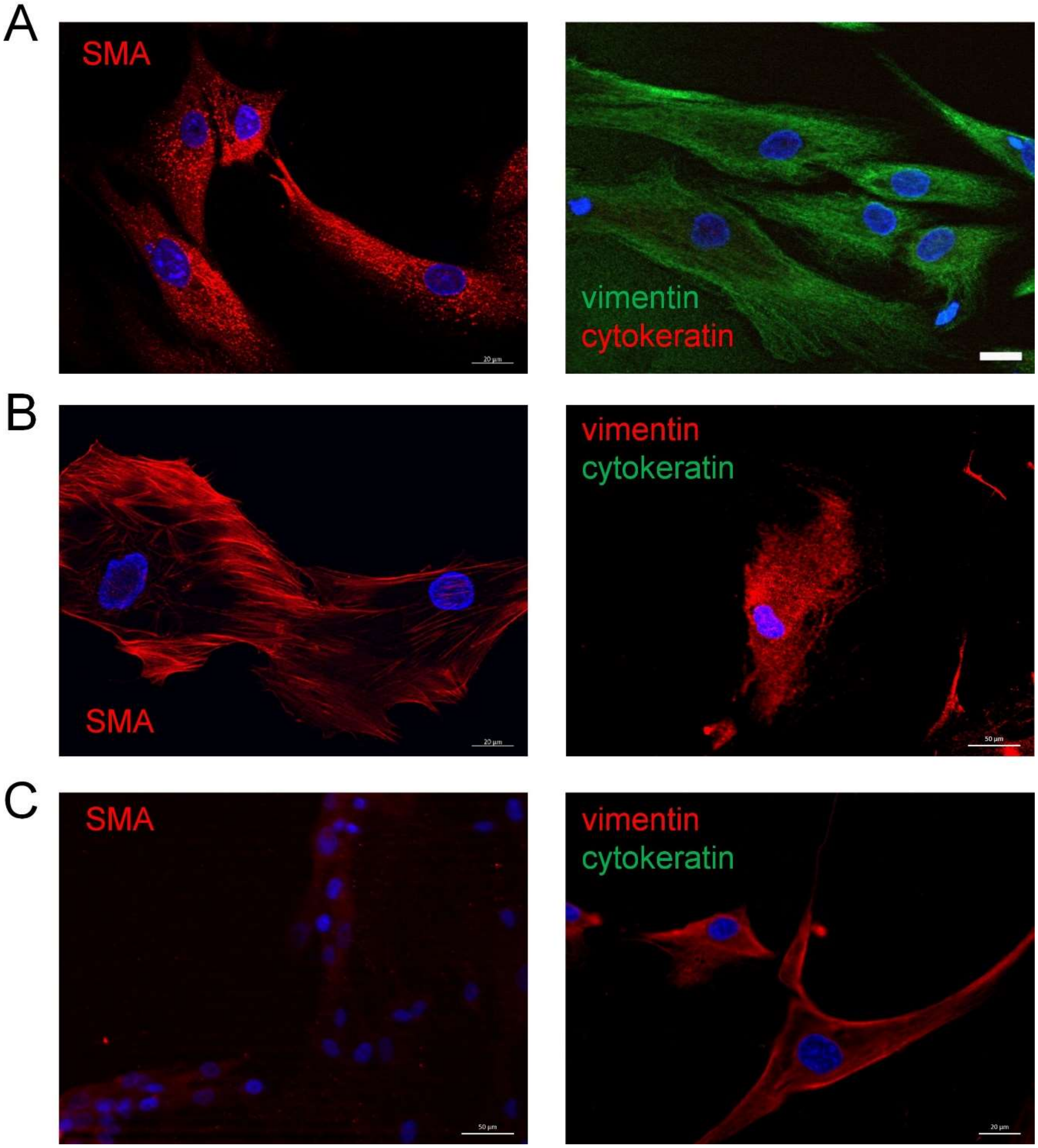

Figure S1. Phenotypic characterization of fibroblasts Submitted as separate file to keep proper resolutions of images.
Representative immunofluorescences of CAF1 (A), CAF3 (B), and NF (E) with antibodies against cytokeratin, vimentin and α-smooth muscle actin (SMA). Scale bars, 20 µm for all except for SMA-NF and vimentin/cytokeratin-CAF3 that are 50 µm.

FIGURE S2

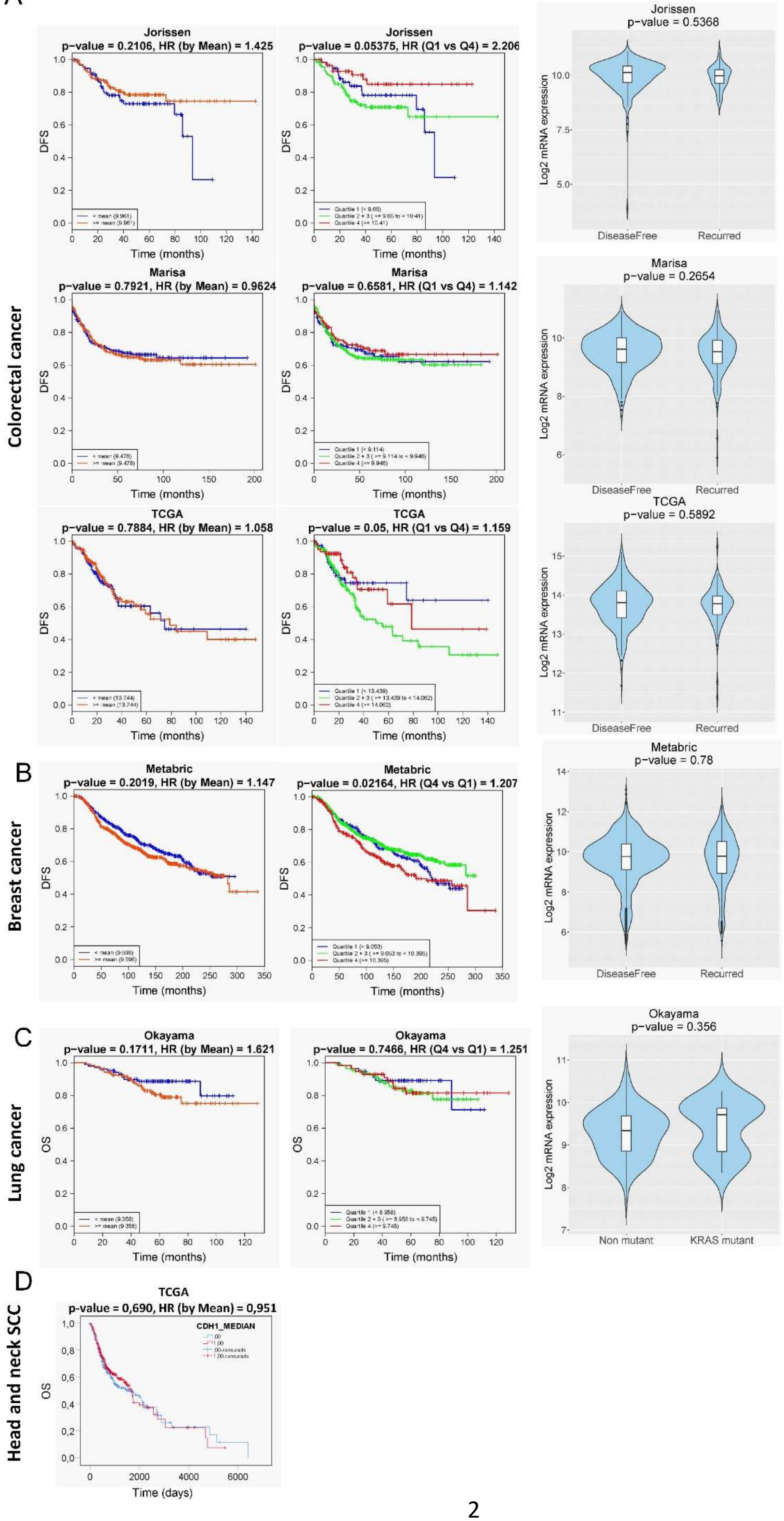
A
B
C
D
Colorectal cancer
Breast cancer
Lung cancer
Head and neck SCC
Jorissen
p−value = 0.2106, HR (by Mean) = 1.425
Jorissen
p−value = 0.05375, HR (Q1 vs Q4) = 2.206
Jorissen
p−value = 0.5368
Marisa
p−value = 0.7921, HR (by Mean) = 0.9624
Marisa
p−value = 0.6581, HR (Q1 vs Q4) = 1.142
Marisa
p−value = 0.2654
TCGA
p−value = 0.7884, HR (by Mean) = 1.058
TCGA
p−value = 0.05, HR (Q1 vs Q4) = 1.159
TCGA
p−value = 0.5892
Metabric
p−value = 0.2019, HR (by Mean) = 1.147
Metabric
p−value = 0.02164, HR (Q4 vs Q1) = 1.207
Metabric
p−value = 0.78
Okayama
p−value = 0.1711, HR (by Mean) = 1.621
Okayama
p−value = 0.7466, HR (Q4 vs Q1) = 1.251
Okayama
p−value = 0.356
TCGA
p-value = 0,690, HR (by Mean) = 0,951
CDH1_MEDIAN
DFS
OS
Time (months)
Time (days)
Log2 mRNA expression
DiseaseFree
Recurred
Non mutant
KRAS mutant

Figure S2. Clinical outcome associated with the E-cadherin expression in head and neck SCCs and other epithelial cancer types. Submitted as separate file to keep proper resolutions of images.
In silico E-cadherin mRNA expression comparative analyses between different groups of patients with colorectal (A), breast (B), lung (C) and head and neck (D) cancer using published datasets. Graphics include Kaplan-Meier curves representing the disease-free survival (DFS) or overall survival (OS) of patient groups selected according to the quartile or mean expression of E-cadherin in the datasets designated as indicated above each graphic. Quartile color code: Q1 (Blue), Q2 plus Q3 (Green), Q4 (Red). Violin plots depict the expression of the E-cadherin among cancer specimens of the indicated group of tumors in the different datasets. Non-mutant and KRAS mutant refer to lung adenocarcinoma specimens with wild type and mutated KRAS, respectively.

Video 1. Representative video showing migration of a spheroid composed of UT-SCC38 cells.

Video 2. Representative video showing migration of a spheroid composed of UT-SCC42B cells.

Video 3. Representative video showing migration of a spheroid composed of UT-SCC40 cells.

Video 4. Representative video showing migration of a spheroid composed of CAF1.

Video 5. Representative video showing migration of a mixed spheroid composed of UT-SCC42B cells and CMFDA-labelled CAF1.

Video 6. Representative video showing migration of a mixed spheroid composed of UT-SCC42B cells and CMFDA-labelled CAF3 cells.
